# Biological Characteristics of Polyurethane-Based Bone-Replacement Materials

**DOI:** 10.3390/polym15040831

**Published:** 2023-02-07

**Authors:** Marfa N. Egorikhina, Andrey E. Bokov, Irina N. Charykova, Yulia P. Rubtsova, Daria D. Linkova, Irina I. Kobyakova, Ekaterina A. Farafontova, Svetlana Ya. Kalinina, Yuri N. Kolmogorov, Diana Ya. Aleynik

**Affiliations:** 1 Federal State Budgetary Educational Institution of Higher Education, Privolzhsky Research Medical University of the Ministry of Health of the Russian Federation, 603005 Nizhny Novgorod, Russia; 2 Limited Liability Company “Icon Lab Gmbh”, 603003 Nizhny Novgorod, Russia

**Keywords:** polyurethane polymer, bone-plastic materials, tantalum pentoxide, zirconium oxide, bismuth oxide, cytotoxicity, fibroblasts, cytocompatibility, adhesion

## Abstract

A study is presented on four polymers of the polyurethane family, obtained using a two-stage process. The first composition is the basic polymer; the others differ from it by the presence of a variety of fillers, introduced to provide radiopacity. The fillers used were 15% bismuth oxide (Composition 2), 15% tantalum pentoxide (Composition 3), or 15% zirconium oxide (Composition 4). Using a test culture of human fibroblasts enabled the level of cytotoxicity of the compositions to be determined by MTT (3-[4,5-dimethylthiazol-2-yl]-2,5 diphenyl tetrazolium bromide) assay, along with variations in the characteristics of the cells resulting from their culture directly on the specimens. The condition of cells on the surfaces of the specimens was assessed using fluorescence microscopy. It was shown that introducing 15% bismuth, tantalum, or zinc compounds as fillers produced a range of effects on the biological characteristics of the compositions. With the different fillers, the levels of toxicity differed and the cells’ proliferative activity or adhesion was affected. However, in general, all the studied compositions may be considered cytocompatible in respect of their biological characteristics and are promising for further development as bases for bone-substituting materials. The results obtained also open up prospects for further investigations of polyurethane compounds.

## 1. Introduction

The proportion of the elderly population is growing, causing a stable global trend of increasing degenerative diseases and increasing frequency of osteoporosis [1,2,3,4,5]. At the same time, a constantly growing number of bone injuries [6,7,8] and of bone cancers [9] are being recorded. Due to these trends, there is a progressively increasing demand for bone replacement materials in the fields of modern traumatology, orthopedics, and oncology. Furthermore, it is impossible not to note the wide use of materials for bone plastics in dentistry and maxillofacial surgery [10,11].

Unfortunately, no osteoplastic material with ideal properties yet exists. Currently, autografts are still the gold standard for osteoplasty [8,12,13], but their use has certain drawbacks (the need for additional surgery, increased risk of infection, insufficient amount of donor material, additional discomfort for the patient). The problems associated with the use of allo- and xenotransplants [14,15] are forcing us to look for alternative materials, and so the development of artificial compounds for osteoplasty is actively continuing.

For implant manufacture, various titanium compounds are widely used, but these have obvious drawbacks despite their good osteoinductive properties, [16] e.g., it has been shown that the elastic modulus of implants made of titanium is significantly higher than that of bone tissue [17,18]. In addition, implants made of this material have high radiodensity values that hinder bone block assessment using CT scanning [19].

Polyetheretherketone (PEEK) is an alternative material used in orthopedics, with mechanical properties close to those of bone tissue [20]. PEEK implant material does not absorb CT radiation and hence does not hinder such assessments of bone tissue condition. On the other hand, PEEK is bioinert and hydrophobic, this making its integration into the bone tissue impossible and promoting a build-up of fibrosis around the implant [18,19,21]. For this reason, the incidence of pseudarthrosis is relatively high after using implants of this material [22,23].

Currently, some of the main artificial materials for bone tissue augmentation are acrylic polymers such as PMMA (poly(methyl methacrylate)). PMMA is a derivative of the acrylic resins developed as plastic materials back in the 1930s. Two components represent the PMMA used for surgery: the liquid monomer and loose powder polymer (with functional additives). Currently, more than 30 different types of bone cements exist developed on the basis of acrylic monomers and polymers. The differences between them are mainly in the ratios of the components used to determine cement fluidity and viscosity.

It has been proven that PMMA bone augmentation is the most effective method for restoring bone support ability, being able to increase the strength of screw fixation in it by 25 to 348 times [24,25,26]. However, this technique has disadvantages due to the physical and chemical properties of PMMA. It is also known that polymerization of this material is an exothermic reaction, resulting in its heating to 50–70 °C. Such high temperatures increase the risk of damaging the surrounding tissues and slowing their regeneration [27,28]. In addition, the cytotoxicity of PMMA has been repeatedly noted, which in turn can negatively affect the regeneration processes [29]. It is thought that methyl methacrylate monomer release may underlie the so-called cement implantation syndrome with possible manifestations such as cardiosuppression and hypotension, sometimes resulting in sudden death in the early postoperative period [30,31]. All of the above makes it relevant to search for a bone-replacement polymer that can be injected without causing chemical/thermal damage to the surrounding tissues or systemic toxic effects.

Today, polymers based on polyurethanes are considered as promising materials for surgery and regenerative medicine [32]. Due to the possibility of their structural modification, polyurethane compounds can have their different physical and chemical properties varied according to the planned application [31]. Their unique properties enable us to obtain both elastic/soft and solid polyurethane-based materials, characterized by good biocompatibility and biostability [31].

The possibility of obtaining a polymer with mechanical properties close to those of bone tissue, good adhesiveness and biocompatibility make polyurethanes promising osteoplasty materials. However, if liquid injection is planned for bone augmentation, it is necessary to ensure effective polymer spreading, usually with the use of fluoroscopic observation. This is achieved by the additional injection of radiopaque contrast agents. Nevertheless, no studies of the effects of such radiopaque contrast agents on the biological properties of polyurethane-based polymers have been published to date.

It should be noted that the interaction of a polymer material with biological objects can determine the success of its biomedical applications. In particular, materials for bone grafting should provide both mechanical strength and create optimal conditions for the vital activity of all the cell populations involved in the process of reparative regeneration, regardless of the localization of the damaged bone [33,34,35,36]. In addition, such materials can act as scaffolding carriers for cells of different origins in the formation of tissue-engineered constructs [37,38,39]. Thus, cytocompatibility, i.e., the positive interaction of the materials being developed with cells, is mandatory to create conditions for tissue repair and for them to be considered promising for subsequent use in clinical practice. The most important aspects of cytocompatibility concern the cellular events occurring directly on the material’s surface. It is necessary to understand how contact with the material and the topography of its surface provide cell adhesion, and affect first of all the cells’ proliferative activity, but possibly also their secretory functions, differentiation potential, etc. [40,41,42]. In particular, the study of the influence of the material on epigenetic regulation of cell gene expression could be one of the most promising research lines in the future [43]. At the same time, characterization of the cytotoxicity of any material being developed is the first and key test in assessing its biocompatibility. This is confirmed by the requirements of international standard ISO 10993-5:2009 (DIN EN ISO 10993-5:2009, German Institute for Standardization, Berlin, Germany), applicable to regulating cytotoxicity preclinical studies in vitro. Further, if the cytotoxicity test is successful, examination of each material prior to preclinical or clinical studies requires continuation of comprehensive cytocompatibility testing in vitro [33,44].

The task of this study was to evaluate the interaction with human cells of an original polyurethane-based material and of various modifications differing in radiopacity, in an in vitro model.

## 2. Materials and Methods

Specimens of four materials of the polyurethane family were investigated. The first and basic material (Composition 1) was a polymer derived from PPG (polypropylene glycol, average molecular weight = 1000), MDI (4,4′-methylene diphenyl diisocyanate) and glycerol; the other materials (Compositions 2, 3 and 4) were modifications of this polymer with radiopaque fillers added. All specimens of the series used in this work were obtained by the same two-stage technology [45]. In the first stage, a polymer was obtained from MDI and PPG. In the second stage of the process, the material was cured by the addition of glycerol as a hardener, using a catalyst dissolved in it (tertiary amines, organotin compounds). During the same stage, one of the radiopaque contrast agents was added—bismuth oxide (Composition 2), tantalum pentoxide (Composition 3), or zirconium oxide (Composition 4). Reagents manufactured by J-S C (Khimreaktiv, Nijni Novgorod, Russia) were used for polymer synthesis. Thus, specimens of materials differing in their radiopaque filler composition were obtained (Table 1).

**Figure 1:**
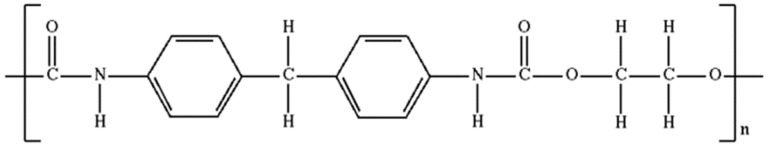
General formula of polymer.

**Figure 2:**
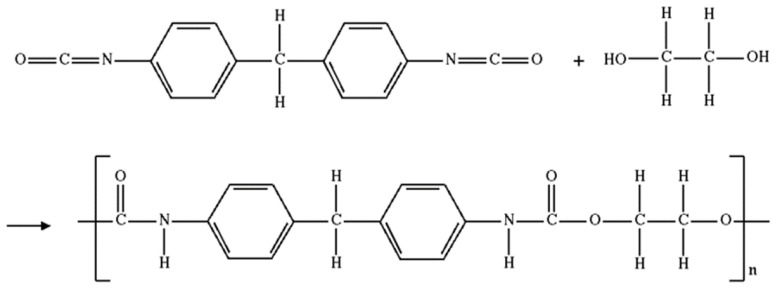
Simplified general formula for polymer production.

After the hardener was added, the curing process of the mixture began, accompanied by a slight heating. In general terms, the polymer can be represented as follows—Figure 1. See Figure 2 for simplified polymer production process.

**Figure 3:**
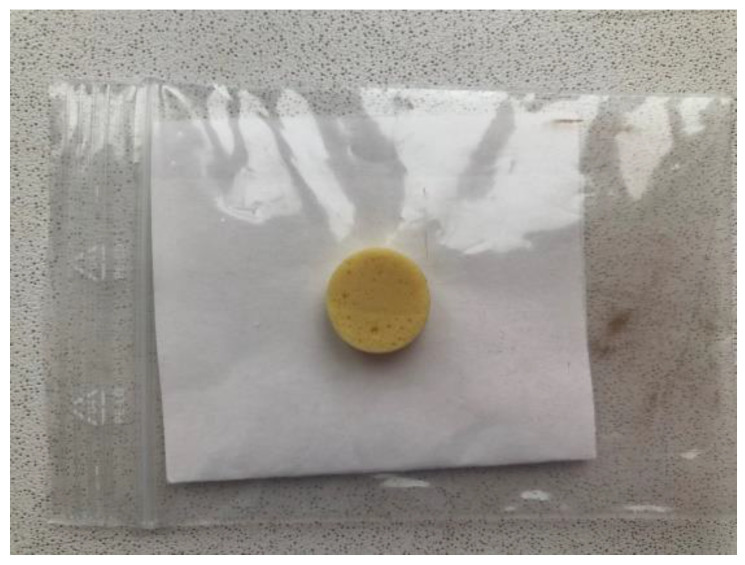
Representative photograph of one of the studied specimens—appearance.

After complete curing, 24 h later, the materials were cut into specimens of the desired shape and size for the study. The specimens of all 4 compositions were sterile yellowish tablets (Figure 3). Each series of 12 specimens was individually labeled and each specimen was individually packaged. The specimens were approximately 10 mm dia × 2 mm thick. Cross sections of the specimens of the materials showed chaotically located holes of different diameters (pores formed during the polymerization reaction); microscopic studies indicated different shapes and sizes of holes distributed across the entire surfaces of the specimens.

To assess the biological characteristics of the presented materials and the possibility of their subsequent application in biomedicine, the level of cytotoxicity and any changes of test culture cells during cultivation on the specimens were evaluated.

To assess the cytotoxicity, an MTT (3-[4,5-dimethylthiazol-2-yl]-2,5 diphenyl tetrazolium bromide) assay [46] was used to determine the estimated relative growth rates (RGRs) of the cells, as percentages based on a determination of the relative optical density (OD) levels. A plate reader was used to run the measurements and to evaluate them according to a ranking scale [47] for each material.

Cell relative growth rate was calculated according to the following formula:

$$\mathrm{RGR}\;\left(\%\right)=\frac{\mathrm{mean}\;\mathrm{OD}\;\mathrm{in}\;\mathrm{the}\;\mathrm{test}\;\mathrm{culture}\;}{\mathrm{mean}\;\mathrm{OD}\;\mathrm{in}\;\mathrm{the}\;\mathrm{control}\;\mathrm{culture}}\times 100$$

where OD is the optical density.

According to the ranking scale [47], the cytotoxicity levels (ranks) of materials represent toxicities as follows: 0 (100% of relative growth rate) and 1 (75–99% of relative growth rate)—nontoxic, 2 (50–74% of relative growth rate)—mild degree, 3 (25–49% of relative growth rate)—medium degree, 4 (1–24 of relative growth rate), and 5 (0% of relative growth rate)—evident toxicity.

The MTT assay test cultures were previously characterized cultures of human dermal fibroblasts obtained in the biotechnology laboratory of the Federal State Budgetary Educational Institution of Higher Professional Education PIMU of the Ministry of Health of Russia. Sampling of biomaterial and obtaining a cell culture with its subsequent use for in vitro studies was approved by the local ethical committee FSBEI HE PRMU MOH Russia (Nizhny Novgorod, Russia) (approved by the local ethics committee on 10 March 2021, protocol No. 5). Cultures of passages 4–6 were taken for studies, with sterility and no contamination with mycoplasmas or viruses confirmed by bacteriological methods and PCR analysis. Cell viability was 97–98% before use in the tests. The culture cells were morphologically homogeneous, predominantly spindle-shaped, less frequently star-shaped with clear contours and prominent outgrowths. The phenotype of the cells corresponded to that of mesenchymal cells: CD 90+, CD 105+, CD 73+, CD10+, CD45−, CD 14−, CD 34−, CD HLA DR−.

The specimens were weighed before testing. The average specimen weight was 156.68 ± 13.67 (130.75 to 184.75). To obtain the extract, multiple specimens were used, weighing in total at least 500 mg for each extraction term (1 or 7 days).

The effects on test cultures of human dermal fibroblasts of the extracts obtained from each material were evaluated by comparing the optical densities of the experimental series and of the control series. The optical density was recorded at 540 nm on a Sunrise analyzer (Tecan Austria GmbH, Grödig, Austria) using Magellan for F50 v7.2 software (Tecan Austria GmbH, Grödig, Austria), allowing for automatic plotting of a calibration curve and determination of the concentrations of the substances under study.

Further, we studied the interaction between the polymer materials and test cells cultivated on their surfaces. During the study, evidence of adhesion to the material surface, variations of cell viability and morphology, and the possibility of cell proliferation on each specimen’s surface during cultivation were evaluated.

To implement this study, a 4–6 passage fibroblast suspension with a density of 10^4^/cm^2^ in 2 mL of complete growth medium was seeded on the surface of each specimen. Each material series was examined in at least three replicates for each experimental period. The specimens were placed in the wells of 24-well culture plates (Costar, Washington, DC, USA). Igla medium modified with Dulbecco (DMEM) and added antibiotics (penicillin/streptomycin), glutamine, and 10% fetal calf serum was used as the growth medium. All media and reagents used were produced by PanEco LLC, Moscow, Russia. Cultivation was run in a humidified CO_2_ incubator atmosphere at 37 °C and 5% CO_2_. The cells were cultured on the specimens for 72 h and culture growth was monitored every 24 h using light microscopy and phase-contrast microscopy (Leica, Wetzlar, Germany, DMI 3000B inverted microscope with LAZ.V.4.3, Leica, Wetzlar, Germany imaging software). After 24 and 72 h of cultivation, part of each specimen was taken for fluorescence microscopy examination, implemented with a Cytation 5 (BioTek, Winooski, VT, USA) imager, allowing for visualization of the cells by phase-contrast and fluorescence microscopy. For the analysis, in vivo cell staining was used with the following fluorochromes: Hoechst 3334 (BD Pharmingen™, Franklin Lakes, NJ, USA) for labeling nuclei; and Calcein AM, BD Pharmingen™ for staining the cytoplasm of viable cells. Staining was performed according to the manufacturers’ protocols.

Fluorochrome Hoechst 3334 (BD Pharmingen™) having high specificity for double-stranded DNA molecules (377 nm excitation wavelength, 447 nm emission wavelength), produced bright blue stained cell nuclei. The use of Calcein AM (469 nm excitation wavelength, 525 nm emission wavelength), resulting in green staining, not only confirmed their viability but also allowed for evaluation of the morphological features and distribution on the specimen surfaces. As a control, growth on the plastic plate (control wells) of the test culture and the variation of its characteristics were evaluated.

The visual images from each specimen were recorded to a video archive, analyzing at least 10 microphotographs from different fields of view.

## 3. Results and Discussion

The level of cytotoxicity is one of the first and most important indicators needed to assess the potential value of such materials for biomedical purposes. The results of our investigation, obtaining extracts after different periods, are presented in Table 2 and Table 3.

The presented materials showed variable cytotoxicity as determined by the MTT assay, e.g., specimen extracts of the base material (Composition 1) and the material containing zirconium dioxide (Composition 4) were nontoxic after both extraction periods, with cytotoxicity ranks of 0–1 (Table 2 and Table 3). The Composition 2 extract containing bismuth showed a mild toxicity level after both periods (rank 2). Composition 3′s toxicity level was variable. Therefore, the 1-day extract showed a cytotoxicity level not exceeding rank 2 when diluted to 1:1 and 1:2. However, the 7-day extract showed a cytotoxicity level corresponding to rank 3, but this was completely leveled when diluted.

**Figure 4:**
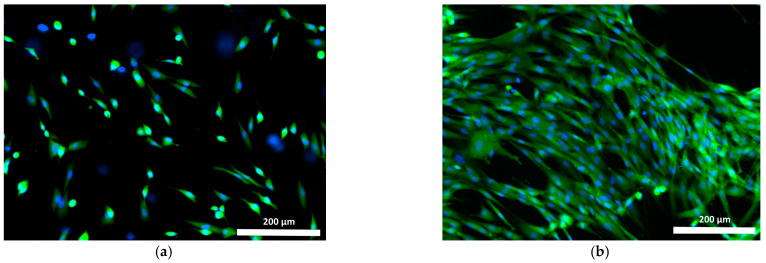
Test culture fibroblasts on plastic (control) (blue stained nuclei—fluorochrome: Hoechst 3334, BD Pharmingen™; green stained cytoplasm of viable cells—Calcein AM fluorochrome, BD Pharmingen™): (**a**) 24 h cultivation; (**b**) 72 h cultivation; mag. 100×.

To study the interaction between cells and the polymer specimens, the cell culture condition was monitored in comparison with that of the test culture seeded on the plastic surface of the culture plate wells. The test culture cells cultured in the control wells were evenly distributed, well-attached, and had spread on the plastic, maintaining their typical spindle shape, or less frequently, a stellate shape with prominent outgrowths throughout the study. By the 72nd hour of cultivation, the culture cells in the control wells had produced confluent monolayers (Figure 4a,b).

**Figure 5:**
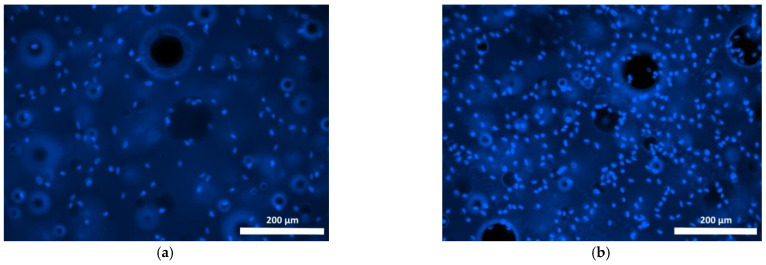
Fibroblast nuclei on the surface of basic Composition 1 specimen (staining fluorochrome—Hoechst 3334, BD Pharmingen™; mag. 100×): (**a**) 24 h cultivation; (**b**) 72 h cultivation.

**Figure 6:**
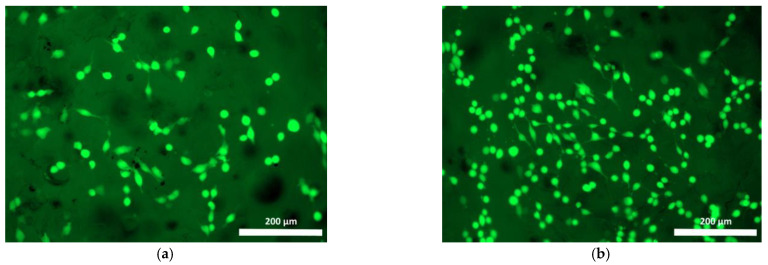
Viable fibroblasts on the surface of specimen of Composition 1 (staining fluorochrome—Calcein AM, BD Pharmingen™; mag. 100×): (**a**) 24 h cultivation; (**b**) 72 h cultivation.

After 24 h of cultivation of the cells seeded on Composition 1 specimens, cell adhesion to the specimen surface could be observed and the cells’ distribution on the specimen surface was fairly uniform (Figure 5a). In the process of further cultivation (after 72 h), the number of stained cell nuclei across the surface of the specimens had visually increased (Figure 5b and Figure 6b). However, we noted that approximately only 50% of the cells were typically spindle-shaped, indicating their good adhesion to the specimen surface (Figure 5a,b). The remaining cells, although attached to the surface of the specimens, retained a spherical shape and did not appear to have spread (Figure 6a,b).

Taking account of the zero cytotoxicity of the base composition, cell attachment to the surface of the specimens, and visual data on cell proliferation on the specimen surfaces, despite the noted incomplete cell proliferation, Composition 1 may be considered as cytocompatible and promising for further application in biomedicine.

As shown above, the basic composition is a polyurethane polymer, and the characteristics we obtained are not in conflict with the properties of similar polymers described by other specialists. Data on the positive biological characteristics of polyurethanes are quite numerous in the literature, e.g., polyurethanes have been shown to have good cytocompatibility and to provide for the attachment and proliferation of mesenchymal stem cells of humans [48], rats [49], and rabbits [50]. Studies on various experimental animals, in turn, testify to the favorable properties of materials based on polyurethanes in respect to enabling bone regeneration processes. This has been confirmed using polyurethanes in experiments on animal models to restore tibial bone defects in sheep [51] and a variety of bone defects in rats [52,53] and mice [54]. However, working with polyurethane compounds requires consideration of the possibility that their biological characteristics may be affected by variations in the composition of the initial components and the catalyst used [55].

It can be assumed that the use of a radiopaque contrast filler, in turn, may also affect the properties of the resulting compounds, and so these will depend to a certain extent on the features of the radiopaque.

Thus, the introduction of bismuth into the polymer composition (Composition 2) led to an increase in cytotoxicity, up to rank 2—mild degree of cytotoxicity, in our specimen extracts obtained from both study periods (Table 2 and Table 3). Furthermore, the 1-day extract retained the same toxicity level (rank 2) even when diluted, this being a possible result of the release into the medium of some kind of rapidly degradable toxic compounds, or perhaps, as quite often occurs, insufficient purification of the original materials. Additionally, the undiluted extract of this specimen, obtained over 7 days, as mentioned above, also demonstrated a toxicity level of 2, although the results for the cytotoxicity of its dilutions varied.

**Figure 7:**
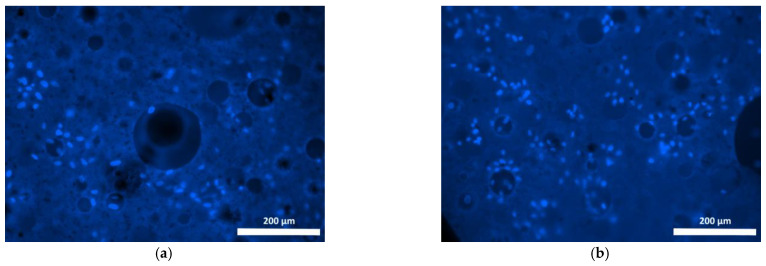
Fibroblast nuclei on the surface of Composition 2 specimen (staining fluorochrome—Hoechst 3334, BD Pharmingen™: (**a**) 24 h cultivation; (**b**) 72 h cultivation. Mag. 100×.

**Figure 8:**
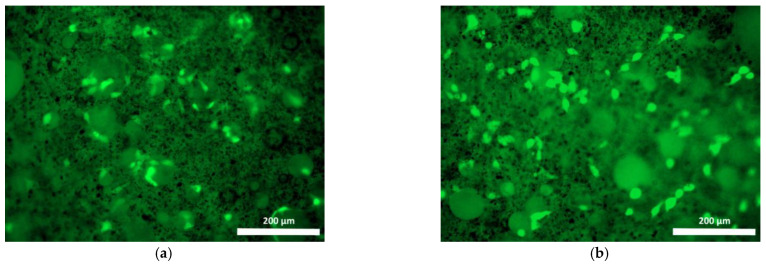
Viable fibroblasts on the surface of Composition 2 specimen (staining fluorochrome—Calcein AM (BD Pharmingen™): (**a**) 24 h cultivation; (**b**) 72 h cultivation. Mag. 100×.

Cells cultured on Composition 2 specimens were distributed less evenly than on the surface of the base composition specimen, mostly being present as small islets (Figure 7 and Figure 8). Use of the fluorochrome Calcein AM (BD Pharmingen™) showed the cells had retained their viability for 72 h, but on the surfaces of these specimens no spread had occurred, neither by day 1 nor by day 3, and the cells had retained their original spherical shapes with short outgrowths (Figure 8a,b). When microphotographs were analyzed visually, the number of cells on the surfaces of these specimens had increased only insignificantly as compared to the cell dynamics on the basic Composition 1. Thus, given that no cell proliferation was evident on the surface of Composition 2 and no cell spread had occurred during the observation period (72 h), these results confirm the data obtained during the specimen’s cytotoxicity study using the MTT assay. Thus, both the one-day and seven-day extracts obtained from specimens containing bismuth oxide showed a mild toxicity corresponding to rank 2.

Bismuth oxide, like other fillers, has been deliberately selected to produce polyurethane-based compositions, given that the value of bismuth compounds has been known in medicine for over 200 years [56,57]. Various drugs containing bismuth have been used in the treatment of syphilis [58] and gastritis, for ulcers [59] (including when the disease was caused by Helicobacter [60,61]), and for other gastrointestinal diseases. Bismuth oxide has been used to manufacture radiopaque rods to repair spinal deformities, and the zero cytotoxicity of these products has been demonstrated in an in vitro model [62]. The antibacterial activity of a number of bismuth compounds is also known [63], and their anticancer effectiveness confirmed [64,65,66]. Obviously, antibacterial activity is often a necessary quality for bone-replacement materials, as inflammation is one of the most common complications after surgical interventions with implants. For this reason, compositions with antibacterial activity, combined with cytocompatibility, would be highly desirable. A study by Shanmugam, Copal, 2014, showed that low concentrations of bismuth compounds are antibacterially active against various microorganisms but were not cytotoxic (rank 1) in their study on murine fibroblasts [47]. However, the same work emphasized that if the bismuth concentration in the material was increased, this would significantly increase the cytotoxic effect. In summary, our data, indicating some limited cytotoxicity of the composition containing bismuth oxide, are, in general, not in conflict with the results of other researchers. The use of materials of cytotoxicity level 2 may be allowed, with certain limitations, when their clinical effect significantly exceeds the clinical risks. In addition, it can be assumed that the composition cytotoxicity may be changed by optimizing the filler concentration.

The cytotoxicity of Composition 3 extracts containing tantalum, obtained after 1 day of extraction, showed rank 1 or 2 (zero to mild degree). The undiluted extract obtained after 7 days’ incubation of Composition 3 specimens showed toxicity of a medium degree (rank 3) (Table 2 and Table 3). The indicated toxicity completely disappeared when the extract was diluted; moreover, all dilutions of this composition extract showed an insignificant stimulatory effect.

**Figure 9:**
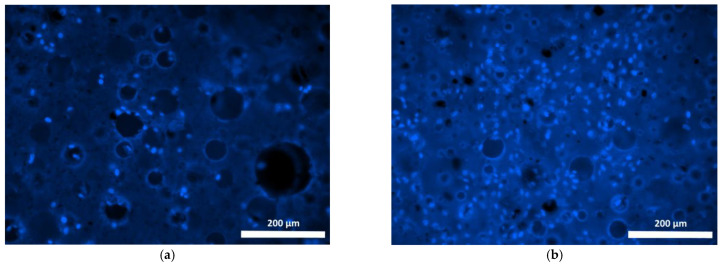
Fibroblast nuclei on the surface of Composition 3 specimen (fluorescence microscopy, fluorochrome—Hoechst 3334, BD Pharmingen™): (**a**) 24 h cultivation; (**b**) 72 h cultivation. mag. 100×.

**Figure 10:**
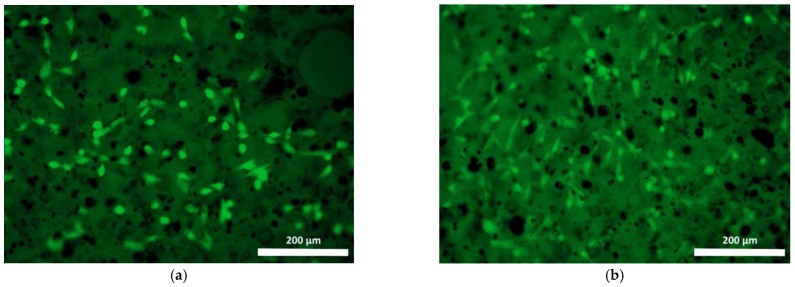
Viable fibroblasts on the surface of Composition 3 specimen (fluorescence microscopy, fluorochrome—Calcein AM, BD Pharmingen™): (**a**) 24 h cultivation; (**b**) 72 h cultivation. mag. 100×.

Test cells cultured on specimens of Composition 3 were distributed unevenly as small colonies. After 24 h, only a small number of spreading cells could be visualized on the surface (Figure 9a and Figure 10a), but by 72 h, many viable, spread cells, typically spindle-shaped and with prominent outgrowths, were already fixed, (Figure 9a and Figure 10b). Thus, the visual characteristics (spread, maintenance of viability, typical morphology, and increase in cell number on the specimens during cultivation) indicated the good adhesion properties of polymer specimens made of the composition containing tantalum.

It should be noted that tantalum and its compounds have a variety of medical applications, including the manufacture of pacemaker electrodes, heart stents, dental implants, artificial joints, radioactive markers, nerve repair nets, hemostasis materials, and dental pulp sealants [67,68,69,70,71,72,73,74,75]. Tantalum is also very attractive for the production of materials for osteoplasty, its biocompatibility being one of the most important characteristics for this purpose. Therefore, the issue of cytotoxicity and cell interaction with tantalum-containing compositions continues to be actively studied, e.g., studies using mouse osteoblasts have shown that low concentrations of tantalum compounds are nontoxic and have a positive effect on the adhesion, proliferation, maturation, and mineralization of osteoblasts [76,77,78,79]. A study by Asadullah et al., 2019, even demonstrated increased cellular responses such as adhesion, proliferation, and differentiation of rat bone tissue mesenchymal stem cells (rBMSCs) when the tantalum content was increased [80].

During the MTT assay, we observed an increase in cytotoxicity of the extract from Composition 3 containing 15% tantalum pentoxide after 7 days of extraction, while the 1-day extract was noncytotoxic (Table 2 and Table 3). The detected increase in toxicity may be attributed to tantalum accumulation in the medium during extraction for 7 days. The results obtained are consistent with the data of Wang et al., 2020, who showed in a study on mouse osteoblasts that the level of toxicity of tantalum compounds may possibly increase if their concentration is increased [81]. At the same time, according to the results of our work, dilutions of the Composition 3 extract activated human fibroblast proliferation as manifested by a clear optical density, and as a consequence, an increase in cell growth relative intensity (Table 3), this being similar to the activation of cell activity by low doses of tantalum, as found in the studies by Wang et al., 2020 [81] and Huo et al., 2017 [76]. These results in combination with the obtained data on good adhesion and the positive dynamics of cell proliferation on the specimen surfaces demonstrate the biocompatibility of Composition 3. In the light of our data, polyurethane compounds with added tantalum may be considered promising for developing basic osteoplasty materials. Studying such compounds in the future, it would possibly be interesting to obtain compositions with different tantalum content and to analyze their biological characteristics.

**Figure 11:**
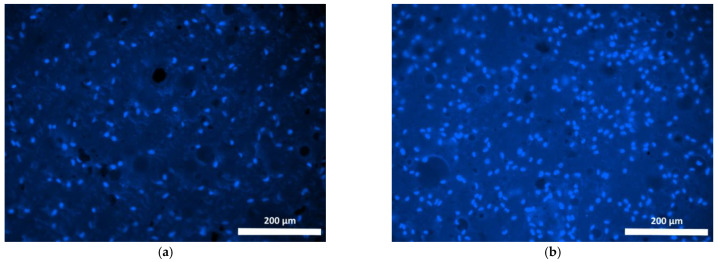
Fibroblast nuclei on the surface of Composition 4 specimen (fluorescence microscopy, fluorochrome—Hoechst 3334, BD Pharmingen™): (**a**) 24 h cultivation; (**b**) 72 h cultivation. Mag. 100×.

**Figure 12:**
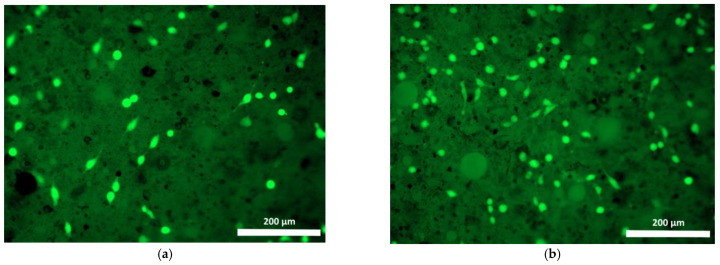
Viable fibroblasts on the surface of Composition 4 specimen (fluorescence microscopy, fluorochrome—Calcein AM (BD Pharmingen™): (**a**) 24 h cultivation; (**b**) 72 h cultivation. Mag. 100×.

Applying the MTT assay to Composition 4 specimens with added zirconium showed that this material was nontoxic (rank 1) at both extraction terms (Table 2 and Table 3). However, when cells were cultured on its surface, the majority of them showed no spread, and no typical adherent morphology was produced within 3 days, and although their distribution was fairly uniform (Figure 11a and Figure 12a), there had been an insignificant increase in the total number of cells on the surface (Figure 11b and Figure 12b).

Furthermore, dilution of the 7-day extract of the zirconium-containing composition showed a positive effect on the proliferation of the test culture cells. However, some inconsistency in the data obtained requires a more detailed study of the zirconium-containing compositions in order to assess their suitability as the basis of osteoplasty materials. The positive qualities of zirconium-based materials, such as their high biocompatibility, mechanical strength, and corrosion resistance, are known [82], and their inclusion into osteoplasty materials is being actively investigated. It has been shown that introducing zirconium compounds into the material structure, while improving its biocompatibility, prevents biofilm formation. Its cause is the evident bactericidal activity of such compounds against a wide range of microorganisms, including various strains of *Staphylococcus aureus* [83,84,85,86]. A number of studies have demonstrated zero cytotoxicity for materials coated with ZrO_2_ [86,87,88,89], e.g., according to Zhang et al., 2012, the cytotoxicity of materials coated with zirconium oxide is no higher than ranks 1 or 2 [89]. Thus, our data are similar to the literature data and confirm the potential value of Composition 4, containing zirconium.

## 4. Conclusions

The presented study demonstrates an investigation of the biological characteristics of polyurethane polymers with different radiopaque filler compositions, when used with human cell cultures. It has been shown that introducing 15% bismuth, tantalum, or zirconium compounds as fillers results in a range of effects on these biological characteristics. In different cases, the level of toxicity is changed, proliferative activity is affected, or cell adhesion is impacted. However, in general, all the studied compositions may be considered cytocompatible in respect of their biological characteristics and are promising for further development as bases for bone-substituting materials.

It should be noted that in addition to the changes in the characteristics of the cells on the surfaces of the studied specimens, the microscopic methods used allowed us to visualize the different distribution and sizes of the pores in their structure, this confirming the potential value of the described materials. Indeed, while the varied effects of the fillers are relevant, the availability of a system of differently sized, interconnected pores appears to be the most important parameter for bone-replacement materials, determining their positive qualities [90,91] and providing for the processes of cell adhesion, subsequent proliferation, and differentiation.

Of course, the data we have presented are preliminary, and a large complex of research lies ahead to develop and study the properties of new polyurethane compounds and their various potential modifications.

## Figures and Tables

**Table 1:** The compositions of the specimens.

Specimen	Specimen Composition
Composition 1	Basic composition: - Standard prepolymer (from MDI and PPG with an average molecular weight of 1000) containing 11–13% of isocyanate groups; - Standard glycerol-based hardener.
Composition 2	Basic composition + 15% bismuth oxide
Composition 3	Basic composition + 15% tantalum pentoxide
Composition 4	Basic composition + 15% zirconium oxide

**Table 2:** Cytotoxicity evaluation of polyurethane polymer specimens at 1 day extraction.

Series	Parameter	Specimen Denomination			
		Composition 1	Composition 2	Composition 3	Composition 4
Control (*n* = 8)	OD	0.451 ± 0.021	0.438 ± 0.018	0.382 ± 0.012	0.433 ± 0.007
	RGR, %	100	100	100	100
	Cytotoxicity level	0	0	0	0
Extract (*n* = 8)	OD	0.414 ± 0.016	0.294 ± 0.018	0.350 ± 0.022	0.403 ± 0.012
	RGR, %	92	67	91	93
	Cytotoxicity level	1	2	1	1
Extract 1:1 (*n* = 8)	OD	0.471 ± 0.015	0.274 ± 0.010	0.279 ± 0.012	0.393 ± 0.011
	RGR, %	104	63	73	91
	Cytotoxicity level	0	2	2	1
Extract 1:2 (*n* = 8)	OD	0.410 ± 0.02	0.298 ± 0.009	0.282 ± 0.009	0.403 ± 0.008
	RGR, %	91	68	74	93
	Cytotoxicity level	1	2	2	1
Extract 1:4 (*n* = 8)	OD	0.452 ± 0.008	0.255 ± 0.009	0.314 ± 0.012	0.374± 0.008
	RGR, %	100	58	82	86
	Cytotoxicity level	0	2	1	1
Extract 1:8 (*n* = 8)	OD	0.372 ± 0.017	0.308 ± 0.008	0.295 ± 0.011	0.392 ± 0.021
	RGR, %	82	70	77	91
	Cytotoxicity level	1	2	1	1

**Table 3:** Cytotoxicity evaluation of polyurethane polymer specimens at 7 day extraction.

Series	Parameter	Specimen Denomination			
		Composition 1	Composition 2	Composition 3	Composition 4
Control (*n* = 8)	OD	0.329 ± 0.008	0.467 ± 0.021	0.330 ± 0.010	0.339 ± 0.010
	RGR, %	100	100	100	100
	Cytotoxicity level	0	0	0	0
Extract (*n* = 8)	OD	0.352 ± 0.008	0.330 ± 0.021	0.126 ± 0.004	0.302 ± 0.006
	RGR, %	107	71	38	89
	Cytotoxicity level	0	2	3	1
Extract 1:1 (*n* = 8)	OD	0.318 ± 0.020	0.525 ± 0.031	0.391 ± 0.011	0.404 ± 0.008
	RGR, %	97	112	118	119
	Cytotoxicity level	1	0	0	0
Extract 1:2 (*n* = 8)	OD	0.259 ± 0.006	0.491 ± 0.035	0.441 ± 0.019	0.447 ± 0.007
	RGR, %	78	105	134	132
	Cytotoxicity level	1	0	0	0
Extract 1:4 (*n* = 8)	OD	0.262 ± 0.007	0.302 ± 0.008	0.362 ± 0.015	0.475 ± 0.021
	RGR, %	80	65	110	140
	Cytotoxicity level	1	2	0	0
Extract 1:8 (*n* = 8)	OD	0.287 ± 0.022	0.292 ± 0.022	0.416± 0.011	0.379 ± 0.049
	RGR, %	87	63	126	112
	Cytotoxicity level	1	2	0	0

## Data Availability

Data presented in this study are available on request from the corresponding author.
